# The role of IRF5 as an immune regulator

**DOI:** 10.1042/BST20250394

**Published:** 2026-09-24

**Authors:** Laura Menocal, Amanda Y.Y. Huang, Anjali Rachelkar, Betsy J. Barnes

**Affiliations:** 1 Center for Autoimmune Musculoskeletal and Hematopoietic Disease, The Feinstein Institutes for Medical Research, Manhasset, NY 11030, U.S.A.; 2 Donald and Barbara Zucker School of Medicine at Hofstra/Northwell, Hempstead, NY 11549, U.S.A.; 3 Elmezzi Graduate School of Molecular Medicine, The Feinstein Institutes for Medical Research, Manhasset, NY 11030, U.S.A.; 4 Department of Pediatrics and Molecular Medicine, Donald and Barbara Zucker School of Medicine at Hofstra/Northwell, Hempstead, NY 11549, U.S.A.; 5 Department of Molecular Medicine, Donald and Barbara Zucker School of Medicine at Hofstra/Northwell, Hempstead, NY 11549, U.S.A.

**Keywords:** adaptive immunity, innate immunity, IRF5

## Abstract

Interferon regulatory factor 5 (IRF5) is a transcription factor at the nexus of immune regulation, inflammation, and autoimmune disease. Studies have shown that dysregulation of IRF5 is strongly implicated in the pathogenesis of autoimmune diseases including systemic lupus erythematosus (SLE), rheumatoid arthritis (RA), and inflammatory bowel disease. While originally described as a regulator downstream of Toll-like receptor (TLR)–MyD88 signaling in myeloid cells, further studies demonstrate a more complex role for IRF5 in mediating adaptive immune responses as well. In this review, we provide a summary of the role of IRF5 across the major immune cell populations in which it is expressed. We discuss the mechanistic basis of IRF5 function in each population, highlight shared themes, and examine the evidence supporting IRF5 as a promising therapeutic target whose inhibition may selectively dampen pathological inflammation while preserving protective immunity. We thus provide an overview of how IRF5 bridges both innate and adaptive immune responses.

## Introduction

The interferon regulatory factor (IRF) family of transcription factors (TFs) comprises nine members (IRF1–IRF9), all involved in mediating immune responses to pathogens and cellular stress. While IRFs were originally reported as mediators of type I interferon (IFN-I) and IFN-stimulated gene (ISG) regulation in response to viral infection, they have also been shown to play critical roles in cell cycle, apoptosis, oncogenesis, and tumor suppression [1–5]. Among these factors, IRF5 has emerged as a critical regulator of antiviral immunity downstream of endosomal toll-like receptor (TLR) activation. Inactive IRF5 primarily resides within the cytoplasm of unstimulated cells. Upon TLR engagement, MyD88 is recruited and initiates a downstream signaling cascade where adaptor protein TASL is recruited by the transporter SLC15A4 and directly binds IRF5. IRF5 undergoes polyubiquitination by TRAF6 and phosphorylation by inhibitor of nuclear factor kappa-B kinase subunit beta (IKKβ). IRF5 then homodimerizes and translocates to the nucleus, where it orchestrates the transcription of various cytokines and chemokines [6–8]. This TASL-dependent step has recently emerged as a potentially druggable node upstream of IRF5, distinct from broader MyD88 signaling. Dysregulation of IRF5 through genetic polymorphisms or aberrant expression has been implicated in the onset and exacerbation of several autoimmune diseases and cancer [9–12]. Recent studies have also correlated nuclear translocation of IRF5 with systemic lupus erythematosus (SLE) severity and relapse [13]. Consistent with its proinflammatory role, *Irf5* deficiency reduces the severity of experimental autoimmune diseases in mice, lowers serum inflammatory cytokine levels, and confers resistance to endotoxic shock [6].

Multiple spliced isoforms of IRF5 exist in humans, each characterized by unique expression and function depending on cell type, cellular and tissue localization, and regulation [14]. Conversely, mice express a single variant of full-length IRF5 transcript, highlighting a limitation of mouse models for studying IRF5 [15]. While initially characterized for its proinflammatory role primarily in myeloid cells [1,16], IRF5 is increasingly recognized for its multifaceted involvement in mediating adaptive immune responses across diverse immune cell types. The role of IRF5 in disease has been extensively reviewed elsewhere [16–18]. Here, we aim to provide a summary of the role of IRF5 across the major immune cell populations in which it is expressed with the goal of synthesizing emerging evidence for IRF5 as a master regulator bridging innate and adaptive immunity.

## Myeloid cells

Myeloid cells are innate immune cells that serve as the first line of defense against immune challenge. Many of these cell types are highly implicated in IFN-I signaling, which IRF5 regulates (Figure 1) [19]. IRF5 in macrophages, monocytes, and neutrophils typically drives inflammation through the production of pro-inflammatory cytokines including tumor necrosis factor alpha (TNF-α), IL-6, IL-12, and IL-23 downstream of the TLR/MyD88/TRAF6 signaling pathway. This leads to IRF5 activation, homodimerization, and subsequent nuclear factor kappa-light-chain-enhancer of activated B cells (NF-kB) activation [20]. Recent studies investigating IRF5 in myeloid populations have suggested more complex roles for IRF5 in pro-inflammatory gene regulation and mechanisms of disease pathogenesis, which we will summarize below.

**Figure 1:**
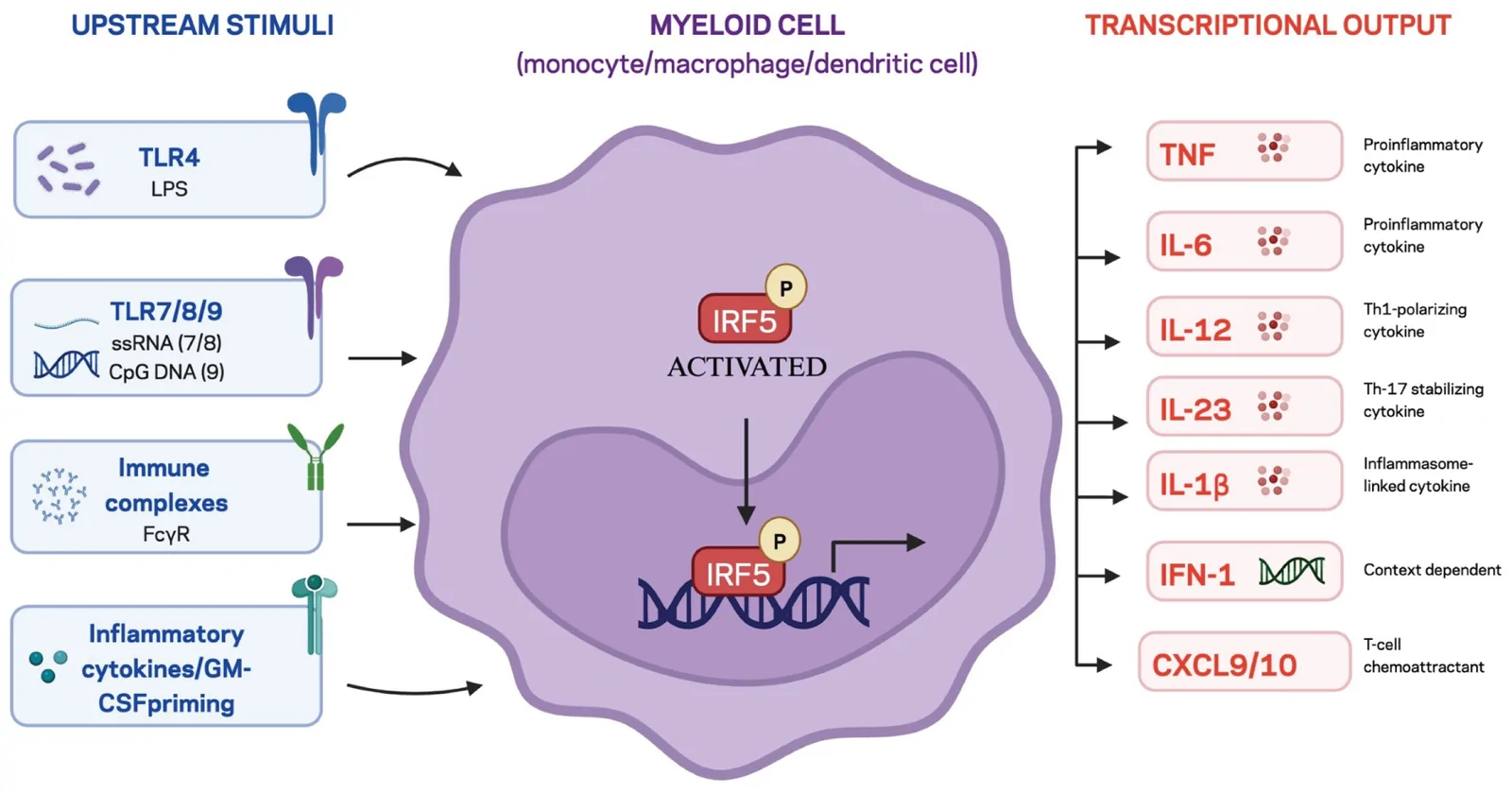
Receptor-mediated IRF5 activation in myeloid cells drives transcription of pro-inflammatory cytokines Receptor-mediated induction of IRF5 phosphorylation, activation, and nuclear translocation induces downstream transcription of various pro-inflammatory cytokines in myeloid cells. Bacterial lipopolysaccharide (LPS) binds TLR4; single-stranded RNA (ssRNA) binds TLR7/8/9; CpG binds TLR9; FcγR mediates signaling downstream of immune complexes, and inflammatory cytokines provide additional priming signals. Ligand engagement triggers phosphorylation (P) of IRF5 in the cytoplasm, promoting its nuclear translocation and binding to target gene promoters. This leads to the transcription of various pro-inflammatory cytokines. Figure created on BioRender.

## Monocytes

Monocytes are a heterogeneous population of bone marrow (BM)–derived mononuclear cells that continuously patrol the circulation upon egress. They are generally categorized into classical, intermediate, and non-classical monocytes, but studies continue to discover more subtypes with diverse roles [21,22]. IRF5 is highly expressed across monocytic subtypes but plays the most significant role in the migration, differentiation, and polarization of classical monocytes [23].

Classical monocytes are the “inflammatory” subset that migrate to tissues upon BM egress, and can further differentiate into tissue-resident, inflammatory (M1), pro-resolving (M2) macrophages, or dendritic cells (DCs) depending on environmental signals [24,25]. In mice, classical monocytes are identified by surface Ly6C^hi^ expression, and in humans, CD14^hi^ expression. At steady state, *Irf5* deficiency does not have a significant effect on Ly6C^hi^ monocyte development, recruitment, or activation [26]. However, a murine model of emphysema demonstrated that IRF5 directly modulates Ly6C expression on monocytes via binding to two regulatory sites within the *Ly6C* promoter [27]. Additionally, it has been shown that during immune challenge, IRF5 activation promotes Ly6C^hi^ monocyte differentiation into M1 macrophages. Using a model of colitis, increased Ly6C^hi^ monocyte recruitment to the gut occurred in wild-type (WT) mice but not in *Irf5*-deficient mice. This work demonstrates that during inflammation, IRF5 is necessary for the influx of monocytes and their differentiation into inflammatory macrophages [26].

Recent studies have implicated a role for IRF5 in classical monocytes in the regulation of other disease models [27–29]. During cigarette smoke exposure, Ly6C^hi^ monocytes in *Irf5*^−/−^ mice down-regulated *Nlrp3* expression, promoting alveolar cell survival and attenuating disease, suggesting that Ly6C^hi^ monocytic IRF5 is crucial in *Nlrp3*-mediated pyroptosis [27]. In the context of influenza A, inflammatory monocytes and monocyte-derived DCs are known mediators of immune response, and IRF5 loss of function led to the reduction in IFN-I released by several cell types in the lungs, including inflammatory monocytes [28]. Similarly, CD14^+^ monocytes isolated from HIV patients showed constitutively higher IRF5 protein and mRNA levels, contributing to the IFN-I response that accompanies the disease [29]. These examples highlight the scope of diseases that monocyte-intrinsic IRF5 plays a significant role in.

Monocyte-intrinsic IRF5 also plays a pivotal role in the pathogenesis of autoimmune disorders including SLE, rheumatoid arthritis (RA), and systemic scleroderma [30–32]. Ly6C^hi^ monocytes are highly associated with SLE due to their IFN-I production, and work using the pristane-induced model of murine lupus identified IRF5 as a major driver of disease. These studies demonstrate that *Irf5*-deficient mice are protected from developing lupus due to decreased expression of CCR2 and CXCR4 in *Irf5*^−/−^ Ly6C^hi^ monocytes, impairing their ability to migrate into the peritoneum [30]. This established the necessity of monocyte-intrinsic IRF5 in regulating chemokine receptor expression and migration during immune challenge, in addition to regulating IFN-I production.

Overall, IRF5 is required for mediating monocytic functions through the regulation of key gene expression. Monocyte-intrinsic IRF5 drives pro-inflammatory polarization through the regulation of Ly6C, migration through the expression of CCR2 and CXCR4, and cell death through NLRP.

## Macrophages

Macrophages are the other crucial component of the mononuclear phagocyte system alongside monocytes. Macrophages are highly plastic and can broadly be categorized as “naïve” M0, “inflammatory” M1 (classical) or “anti-inflammatory” M2 (alternative) [33]. IRF5 drives M1 polarization by binding to the promoter regions of M1-associated genes, leading to inflammatory cytokine release and anti-pathogen responses [34–37].

IRF5 orchestrates several critical functions within M1 macrophages. First, it is essential for maintaining plasma membrane structure of M1 macrophages [38]. Silencing of IRF5 in M1 macrophages leads to membrane restructuring and increased stiffness, resembling M2 macrophages. Macrophage-intrinsic IRF5 is also required for bacterial clearance through the induction of the iNOS pathway [35,39]. Furthermore, IRF5 regulates genes involved in reactive oxygen species (ROS) production and autophagy, such as *p40phox* and *ATG5*, both vital for antimicrobial defense. Beyond direct M1 functions, IRF5 also critically impacts inflammatory diseases. In aortic adventitial aneurysm (AAA), an inflammatory condition characterized by aortic wall thickening mediated by macrophage infiltration, macrophage-intrinsic IRF5 was demonstrated to regulate migration to the adventitia by controlling *Pik3cg* expression in mice [40].

IRF5 is an emerging regulator of macrophage metabolic processes. ChIP- and ATAC-sequencing of airway macrophages exposed to influenza A revealed that *Irf5^−/−^* macrophages were more oxidative than wild-type cells, mimicking M2 macrophages. This highlights a role for IRF5 in the regulation of glycolysis in macrophages [41]. IRF5 in adipose tissue macrophages is also metabolically significant. During caloric excess, such as obesity, it suppresses *Ghitm* expression, a component of the mitochondrial matrix [42]. *Ghitm* suppression leads to abnormal mitochondrial cristae structure. This reprograms macrophages to modulate metabolic programming in favor of glycolysis over oxidative phosphorylation, leading to increased inflammatory glycolytic products and M1 polarization.

IRF5 dysregulation in macrophages has been linked to autoimmune and inflammatory diseases. One major inflammatory condition that arises from rheumatic disease (such as sJIA and SLE) and EBV infection is macrophage activation syndrome (MAS). In a study utilizing a TLR-driven mouse model of MAS, it was found that IRF5 drives the differentiation of Ly6C^hi^ monocytes into a specialized population of hemophagocytic macrophages that are responsible for the development of cytopenias and inflammatory cytokine production [43]. Further work is needed to determine the mechanism behind IRF5-driven hemophagocyte differentiation.

## Microglia

Microglia are resident macrophages of the central nervous system (CNS) that exist on a spectrum of subtypes [44–46]. Ramified microglia are “resting” and mainly function to surveille the CNS. M1-like microglia adopt an “ameboid” morphology, become highly phagocytic, and release inflammatory cytokines. M2-like microglia exert neuroprotective functions, including the release of anti-inflammatory cytokines and mediating tissue repair [47,48]. Consistent with its role in various other cell types, IRF5 holds the most significant role in activated, inflammatory microglia, which are mainly present during immune challenge [47].

The role of IRF5 in microglia has been explored in neurodegenerative and neuroinflammatory conditions, including Alzheimer’s, ischemic stroke, and amyotrophic lateral sclerosis (ALS) [12,49,50]. In most cases, IRF5 plays its canonical role in signaling through MyD88/TRAF6/NF-kB, where it drives polarization to an inflammatory state and promotes the release of TNF-α, IL-6, and IL-12 [51,52]. Microglial-intrinsic IRF5 is also responsible for producing cytokines that drive neuronal apoptosis in a murine ALS model [12]. *Irf5* silencing resulted in decreased expression of apoptotic markers BAX and cleaved caspase-3, and increased BCL-2, emphasizing the importance of IRF5 in regulating microglial cell death.

A non-canonical role of IRF5 was discovered in a study utilizing the experimental autoimmune encephalitis (EAE) murine model of multiple sclerosis (MS) [53]. Transcriptomic analysis of *Irf5*^−/−^ microglia revealed down-regulation of genes associated with GTPase activity, lipid metabolism, and cholesterol biosynthesis, which aligned with their inability to properly remyelinate during the recovery phase of EAE. *Irf5*^−/−^ microglia also had increased phagocytic activity but were unable to properly degrade lipid droplets. Accumulation of lipid droplets led to the development of foamy microglia that drive a proinflammatory state, potentially explaining the exacerbation of EAE in the recovery phase of *Irf5*^−/−^ mice. This study unraveled a new, instrumental role of IRF5 in lipid homeostasis in the context of neurodegeneration.

## Neutrophils

As the most abundant granulocytes, neutrophils are critical first-line innate immune cells, mediating host defense through phagocytosis, cytokine release, and NETosis [54,55]. While IRF5 is not necessary for neutrophil development, it is important for immune responses [56]. In a bacterial peritonitis model, low-dose bacterial challenge induced IRF5 up-regulation in neutrophils, mediating a robust secondary immune response. This up-regulation correlated with enhanced IFN-g production and TNFR1 expression [57]. mRNA-seq analysis of murine HoxB8 neutrophils stimulated with zymosan identified that IRF5 controls the expression of target genes involved in neutrophil activation and migration (*Cav1*), bacterial phagocytosis, ROS production, and neutrophil extracellular trap (NET) formation [58]. Using a murine model of arthritis, it was demonstrated that mice lacking *Irf5* had decreased neutrophil accumulation in knee joints relative to WT mice [59]. This was due to decreased levels of neutrophil-chemoattractant CXCL1 rather than a neutrophil intrinsic defect. While IRF5 is clearly essential for multiple neutrophil effector functions, particularly driving inflammatory responses, the precise contributions of neutrophil-intrinsic IRF5 require further elucidation.

## Basophils

Basophils, the rarest granulocytes in circulation, drive robust IgE-mediated type-2 immune responses through the production of histamine, IL-3, IL-4, and IL-13 [60]. While IL-3 enhances basophil function, IFN-I signaling negatively regulates basophil responsiveness to IL-3 but does not impair their degranulation ability [61,62]. Mass cytometry analysis has shown high IRF5 expression in human lung basophils, though its direct role in basophil function is yet to be uncovered [28]. Interestingly, a mouse model of renal fibrosis identified a specialized CXCR2^+^ basophil population capable of producing IL-6 and inducing T_h_17 differentiation. This raises the possibility of IRF5 involvement, as IRF5 is known to promote the production of T_h_17-associated cytokines in T cells [63,64].

## Eosinophils

Eosinophils are highly cytotoxic granulocytes that mediate host defense against parasites, contribute to inflammation, and are associated with disease pathogenesis [65]. A study on the role of IRF5 in allergy responses demonstrated that IRF5 negatively regulates allergy-associated T_h_2 responses, including impaired eosinophil recruitment to the lungs, assessed by bronchoalveolar lavage samples [66]. Further work is needed to establish the role of eosinophil-intrinsic IRF5 in allergy and autoimmunity.

A more direct link between IRF5 and eosinophil activity was established in a colorectal cancer study, finding that GM-CSF induction by IRF5 increases eosinophil antitumor activity [67]. Utilizing the MC38 colorectal cancer cell line in Eo-cre *csf2rb*^fl/fl^ mice, they found that GM-CSF signaling in eosinophils was crucial for controlling tumor burden, indicating that this signaling pathway is necessary for eosinophil tumoricidal activity. RNA sequencing of GM-CSF treated BM-derived eosinophils identified *Irf5* as a gene of interest, and tumor-infiltrating eosinophils in MC38 tumors displayed increased IRF5 activation. Eo-cre *Irf5*^−/−^ mouse tumor burden phenocopied Eo-cre *csf2rb*^fl/fl^ mice, highlighting the potential of targeting eosinophil-intrinsic IRF5 to combat cancer [67].

## Dendritic cells

DCs are a heterogenous subset of myeloid cells that are at the forefront of initiating and bridging innate and adaptive immune responses. Broadly, they can be classified as classical DCs (cDC1s and cDC2s), plasmacytoid DCs (pDCs), and monocyte-derived DCs (moDCs), each exhibiting distinct surface markers and functions [68]. While IRF4 and IRF8 are the TFs best associated with DC development, IRF5 is also highly expressed in DCs [69]. Interestingly, human-induced pluripotent stem cells lacking *Irf5* successfully differentiate into mature cDC, indicating that IRF5 is dispensable for DC development and instead acts downstream on differentiated cells [28].

In pDCs, IRF5 plays only a partial role in the production of IFN-I, as it predominantly controls the production of IL-12B, IL-6, and TNF-α [6,70]. This subset specificity was also demonstrated in patients with SARS-CoV-2 infection, where IRF5 expression did not correlate with IFN-α production in pDCs, despite its association with pro-inflammatory output in other DC populations [71]. Interestingly, reduced IRF5 expression in moDC correlated with reduced circulating DC populations, supporting a role for IRF5 in the maintenance of inflammatory DC responses during viral infection [71]. IRF5 expression was also shown to correlate with increased expression of the inhibitory receptor CD85k (ILT-3) on myeloid DCs, suggesting that IRF5 regulates both inflammatory and immunomodulatory programs [71].

A similar dual capacity for IRF5 as a transcriptional activated and repressor is evident in both conventional and bone-marrow derived DCs (BMDCs). In West Nile virus infected mouse BMDCs, lack of *Irf5* altered NF-κB associated inflammatory programs, including dysregulated induction of TLR7, TLR8, and inflammasome-related genes such as *Aim2*, *Pycard*, *Nlrp10*, and *Il-18* [72]. In parallel, IRF5 WT DCs showed down-regulation of cytokine related genes *IL-1b*, *IL20rb*, and *Cd14* expression, suggesting that IRF5 can function both as a transcriptional activator and repressor within DCs. In the context of *Toxoplasma gondii* (*T. gondii*) infection, *Irf5*-deficient mice exhibited reductions in IL-12 and IFN-γ responses and increased susceptibility to *T. gondii* infection [73]. Beyond inflammatory cytokine production, it was demonstrated that myeloid-cell intrinsic IRF5 regulates multiple checkpoints required for effective CD4^+^ T cell responses. *Irf5*-deficient BMDCs induced weaker CD4^+^ T cell proliferation, reduced IL-2 production, impaired T_h_1 and T_h_17 differentiation, and increased T_h_2/Treg associated responses, suggesting that IRF5 acts as a checkpoint linking DC function to T cell fate [74].

Taken together, these findings across different DC subsets suggest that IRF5 doesn’t act solely as a pro-inflammatory regulator, but exhibits distinct, subset-specific roles in DC biology and disease context.

## Lymphoid cells

Lymphoid cells are adaptive immune cells that confer antigen-specific responses, as well as immune memory. They are comprised of T cells, which develop in the thymus, and B cells, which develop in the BM. While IRF5’s role in lymphocytes is less classically defined than in myeloid cells, several lymphocyte subsets are implicated in IFN-I signaling and antibody-mediated immunity, processes in which IRF5 plays a regulatory role. In B cells, IRF5 acts downstream of B cell receptor (BCR)/TLR9 receptor engagement to shape class switching, plasma cell differentiation, and the generation of age-associated B cells (ABCs) [75–77]. In T cells, IRF5’s roles are less classically defined but include roles in shaping T-cell differentiation and effector responses [63,78]. Recent studies investigating IRF5 in lymphoid cells reveal context-dependent and cell specific mechanisms of regulation, which we will highlight below.

### CD4^+^ T cells

CD4^+^ T cells are a heterogeneous population of adaptive immune cells whose differentiation and functional polarization depends on cytokine signaling, antigenic stimulation, and the expression of lineage-defining TF [79]. In naïve CD4^+^ T cells, IRF5 is expressed at relatively low levels but increases in a time-dependent manner post-activation *in vitro* [63]. Recent evidence highlights IRF5 as a regulator in shaping CD4^+^ T-cell fate and effector programs (Figure 2). In a murine model of colitis, *Irf5^−/−^* mice were protected from immune pathology and exhibited a decrease in the accumulation of T_h_1 cells [26]. Similarly, in a model of *Leishmanania donovani* infection, hosts systemically lacking *Irf5* exhibited impaired Th1 development and responses [80]. Mechanistically, loss of *Irf5* impaired downstream T cell receptor (TCR) signaling and resulted in a *Tbet-*mediated decrease in the production of pro-inflammatory cytokines associated with T_h_1 and T_h_17 responses, including IFN-γ and IL-17. Instead, there was an increase in T_h_2 responses [63,78]. A recent study using a murine model of EAE demonstrated that *Irf5* regulates protein translation in CD4 T cells via mTORC1 signaling, and that mice lacking *Irf5* were protected from disease pathogenesis [81].

**Figure 2:**
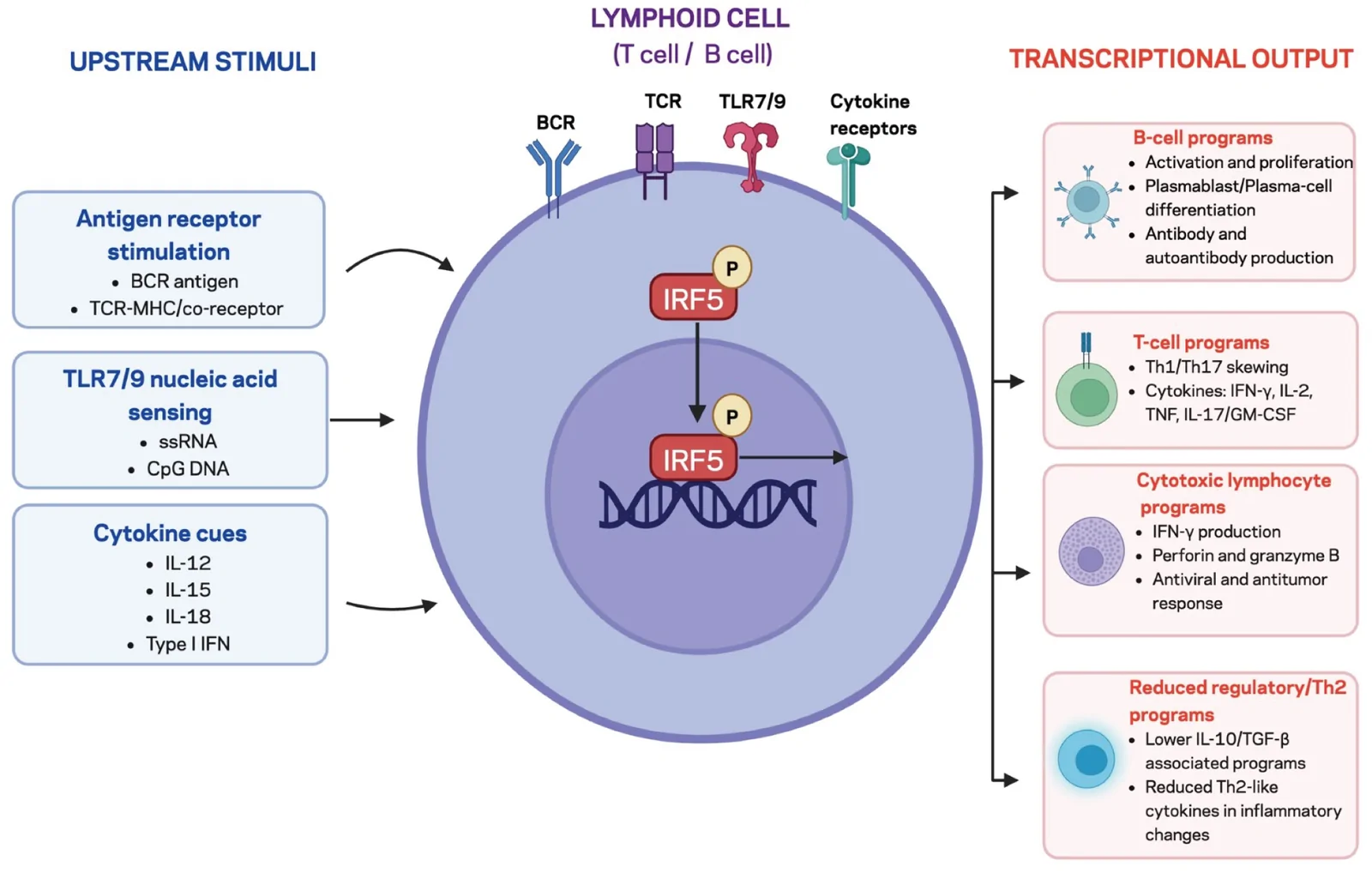
Receptor-mediated activation of IRF5 in lymphoid cells results in varying context-dependent transcriptional outputs Upstream cues, including antigen receptor stimulation, TLR7/9-mediated nucleic acid sensing, costimulatory signals, and inflammatory cytokine signaling, phosphorylate and activate IRF5, which translocates to the nucleus and regulates target gene expression in B cells and T cells. In lymphoid cells, IRF5 controls effector programs in a context-dependent manner. Figure created on BioRender.

Interestingly, follow-up *in vitro* studies using cells from T cell-specific *Irf5* conditional knockout mice revealed that initial findings from global *Irf5^−/−^* mice, which suggested a T cell-intrinsic role, were more likely attributable to defects in *Irf5*-deficient antigen-presenting cells (APCs) rather than T cells themselves [82]. Despite this, further investigation highlighted a distinct role for IRF5 in CD4^+^ T cells downstream of TLR7, leading to the up-regulation of death receptor 5 (DR5). This mechanism ultimately drives CD4^+^ T cell death during chronic infection, such that mice lacking T cell-specific *Irf5* exhibited increased frequencies of IFN-γ producing CD4^+^ T cells [82]. Supporting this, analyses of HIV-infected patients showed increased IRF5 expression in IFN-γ positive CD4^+^ T cells, correlating with enhanced susceptibility to Fas-mediated apoptosis, which could be inhibited by IRF5-blocking peptides [83].

In addition to its role in T_h_1/T_h_17 responses, IRF5 was recently implicated in Tfh biology. It was demonstrated that *Irf5^−/−^* mice exhibit a defect in the ability to produce functional Tfh cells to properly generate germinal centers (GC) in response to T cell-dependent immunization [77]. Given that Tfh support B cell maturation, affinity maturation, and antibody production, these findings suggest that IRF5 may contribute more broadly to adaptive immune responses. The exact mechanisms by which IRF5 regulates Tfh development and function remain to be elucidated.

T_h_2 cells are a subset of CD4^+^ T cells that mount a response against helminth infections, allergies, and tissue repair. In contrast with its role in T_h_1 and T_h_17 inflammatory responses, IRF5 is actively suppressed during T_h_2 differentiation by IRF4 [84]. This agonistic dynamic between IRF4 and IRF5 may represent an important transcriptional checkpoint governing CD4^+^ T-cell differentiation and fate commitment.

### CD8^+^ T cells

CD8^+^ T cells are cytotoxic lymphocytes that upon proper priming and activation mediate effector function through the secretion of effector cytokines (IFN-γ and TNF-α) and lytic molecules (perforin and granzymes). Although the role of IRF5 in CD8^+^ T cells is less well characterized than in myeloid cells or CD4^+^ T cells, emerging evidence highlights a functional role for IRF5 in CD8^+^ T-cell activation and differentiation (Figure 2). Like in CD4^+^ T cells, IRF5 is expressed at relatively low levels in naïve CD8^+^ T cells, and its expression increases with activation over time *in vitro* [85]. IRF5 has been shown to regulate CD8^+^ T-cell function through regulation of mitochondrial metabolism. Chronic infection studies using Lymphocytic choriomeningitis virus clone 13 (LCMV Cl13) demonstrated that *Irf5* deficiency results in defective lipid metabolism, increased ROS, and reduced oxidation phosphorylation, leading to increased cell death [85]. In a melanoma model using both whole-body and CD8^+^ T cell-specific *TOPK* knockout mice, it was demonstrated that CD8^+^ T cell effector function is dampened by TOPK-mediated IRF5 inhibition, resulting in rapid tumor growth [86]. Suppression of IRF5 was associated with dampened cytotoxic capacity. This work was further validated using primary human CD8^+^ T cells and overall suggests that IRF5 plays an important role in mediating anti-tumor effector activity.

Interestingly, IRF5 expression was high in a novel CD86^hi^ “regulatory” CD8^+^ tumor-infiltrating lymphocyte population within a melanoma mouse model [87]. This suggests that IRF5 may contribute to the development of distinct functional CD8^+^ T cell subsets within the tumor rather than just effector populations. Given the emerging evidence of distinct T-cell subsets in solid tumors, further investigation on how IRF5 expression differs among these subsets is warranted [88].

Beyond cancer, IRF5 has been implicated in CD8^+^ T-cell responses to infectious disease. In a murine model of *Leishmania donovani* infection, antigen-specific CD8^+^ T cells exhibited limited expansion, and this was mediated by DC-intrinsic IRF5 signaling and hypoxia-inducible factor 1-alpha (HIF-1α) -dependent pathways [89,90]. These findings highlight the fact that IRF5 influences adaptive immune responses both intrinsically and extrinsically through modulation of myeloid cell function.

Collectively, current evidence suggests that IRF5 contributes to several aspects of CD8^+^ T-cell biology, including activation, expansion, and effector function. However, future studies utilizing conditional *Irf5* deletion models as well as single cell sequencing approaches would be advantageous to fully elucidate the role of IRF5 in shaping CD8^+^ T-cell fate, persistence, and memory formation in various disease contexts.

## B cells

B cells are the antibody-secreting cells of the adaptive immune system. IRF5 is known to play a crucial role in B cell biology, especially in the context of autoimmunity. Murine studies have established that IRF5 is dispensable for B cell development, as *Irf5^−/−^* mice exhibit normal B cell development, but is necessary for activation-induced effector function [6,77].

IRF5 nuclear translocation and activation in B cells occurs predominantly downstream of TLR9 signaling, and is required for B cell proliferation, plasma cell differentiation, and IgG class switching and secretion in response to stimulation [91]. It has also been shown to be important for GC formation, as loss of one allele significantly reduces GC formation [92].

Beyond its role in GC responses, IRF5 has been shown to be a key regulator of ABCs, a distinct subset that can be differentiated by expression of CD11c and *Tbet*, and that arises prematurely in autoimmune disease [76,93]. In a B cell-specific IRF5 knockout murine lupus model, it was demonstrated that IRF5 regulates IL-21-mediated differentiation of ABCs [76]. This appears to be a mechanistically distinct role for IRF5 relative to its role in GC plasma cell formation. Adding further nuance, recent work employing *Irf5*^−/−^ mice revealed that IRF5’s regulation of plasma cell formation is context-dependent, varying between T cell dependent and TLR-dependent stimulation [77].

Notably, these TLR9-mediated findings were not replicated when *Irf5^−/−^* B cells were transferred into *Rag2^−/−^* mice, suggesting that TLR9-dependent, IRF5-mediated plasma cell regulation may not be entirely B cell-intrinsic. Consequently, these studies indicate that at least a portion of IRF5-dependent plasma cell responses are regulated through B cell-extrinsic mechanisms, potentially involving Tfh cells or DCs. Consistent with the ongoing role for IRF5 in disease, a tamoxifen-inducible *Irf5*-deficient mouse model revealed that ablation of *Irf5* after disease onset reduced disease severity in SLE [94,95].

Evidence for the role of IRF5 in B cell-mediated autoimmunity has extended beyond mouse models. Studies using primary human B cells demonstrated that IRF5 is expressed across all B cell subsets (naïve, transitional, memory, plasma cells, and plasmablasts) [75]. Notably, the downstream targets of IRF5 include *Irf4*, *Erk*, and *Myc*, which are distinct from those identified in murine models, highlighting species-specific differences in how IRF5 governs B cell intrinsic programs [75]. Genetic studies have further implicated IRF5 to B cell-driven pathology in SLE. A recent human study involving patients with SLE demonstrated that not only does expression of IRF5 correlate with disease severity, but that nuclear translocation can serve as a biomarker for disease activity and flare-ups [13].

Given that SLE and other autoimmune diseases disproportionately affect women, a direct link between IRF5 and sex-specific B cell has also been established. Healthy women were shown to have a higher frequency of IRF5^+^ B cells than men, both at baseline and following TLR7 or TLR9 stimulation *in vitro* [96]. Functionally, TLR-stimulated B cells from women produced higher levels of TNF-α than those from men. Collectively, these data support a model where IRF5 serves as an important regulator of activation-induced B cell responses in both mouse and humans.

## Conclusion

Here, we have summarized the current knowledge on the role of IRF5 in immune cell-specific regulation. In myeloid cells, IRF5 is a prominent driver of proinflammatory cytokine production, macrophage polarization, migration, and immune cell recruitment. It also contributes to specialized processes including hemophagocyte differentiation and lipid homeostasis in microglia. In the adaptive compartment, IRF5 influences CD4^+^ T-cell fate decisions by promoting T_h_1 and T_h_17 polarization. In CD8^+^ T cells, it governs lipid metabolism and mitochondrial function to prevent exhaustion during chronic infection. In B cells, IRF5 regulates activation, expansion, and plasmablast differentiation. Taken together, these findings support a model where IRF5’s functions are not restricted to innate cells but rather are more involved in orchestrating inflammatory responses across *both* innate and adaptive immune systems.

Despite significant advances in understanding the multi-faceted roles of IRF5, many questions remain. For example, the precise mechanisms by which IRF5 regulates basophil, eosinophil, and neutrophil effector programs are still largely unexplored. Additionally, the transcriptional programs required for IRF5-mediated GC reactions, memory formation, and autoreactive B cell formation remain incompletely understood. In T cells, the role of IRF5 in differentiation in the context of acute and chronic antigen stimulation also remains fully unexplored, as well as how IRF5 expression regulates different T cell subsets, including memory and exhausted T cells. To date, most studies have been conducted *in vitro* or using whole-body *Irf5* knockout mice. Future work should incorporate the use of cell-specific conditional knockout mice to perform kinetic studies on the temporal regulation of IRF5 at homeostasis and in the context of disease, as well as single cell multi-omics.

The growing appreciation of IRF5 as a transcriptional regulator bridging innate and adaptive immunity, coupled with its strong genetic associations with various diseases, positions it as a promising therapeutic target. Continued efforts to define the cell-specific and context-dependent functions of IRF5 will be necessary not only for advancing our fundamental knowledge of immune regulation but also for the development of targeted therapies.

## Perspectives

- IRF5 plays a critical role in mediating inflammatory responses in both autoimmunity and cancer.
- IRF5 has been predominantly characterized in the context of innate immune responses, specifically myeloid cells. Its overexpression is associated with hyperactive immunity. While originally characterized in myeloid cells, emerging evidence demonstrates an existing role in governing effector programs in adaptive immune cells as well.
- Understanding cell-specific roles of IRF5 in *both* cell and disease-specific contexts will aid in the development of more targeted therapies.

## Open Access

Open access for this article is provided via our Subscribe to Open programme, made possible through the support of our subscribing institutions. Learn more here: https://portlandpress.com/pages/open_access_options_and_prices#conditional.

## Abbreviations

AAA: aortic adventitial aneurysm
ABC: age-associated B cell
ALS: amyotrophic lateral sclerosis
APC: antigen-presenting cell
ATAC-seq: Assay for transposase -accessible chromatin-sequencing
BCR: B-cell receptor
BM: bone marrow
BMDC: bone marrow-derived dendritic cell
CCR2: C–C motif chemokine receptor 2
ChIP: chromatin immunoprecipitation
CNS: central nervous system
DC: dendritic cell
EAE: experimental autoimmune encephalitis
EBV: Epstein–Barr Virus
GC: germinal center
HIV: human immunodeficiency virus
HIF-1α: hypoxia-inducible factor 1-alpha
IFN-1: Type 1 interferon
IKKβ: inhibitor of nuclear factor kappa-B kinase subunit beta
IRF5: interferon regulatory factor 5
IL: interleukin
IRF: interferon regulatory factor
ISG: interferon-stimulated genes
LCMV: lymphocytic choriomeningitis
MAS: macrophage activation syndrome
Mo-DC: monocyte-derived DC
MS: multiple sclerosis
NET: neutrophil extracellular trap
NF-kB: nuclear factor kappa-light-chain-enhancer of activated B cells
RA: rheumatoid arthritis
ROS: reactive oxygen species
SLE: systemic lupus erythematosus
TCR: T-cell receptor
TF: transcription factor
Tfh: T follicular helper
Th: T helper
TLR: Toll-like receptor
TNF: tumor necrosis factor
TNF-a: tumor necrosis factor alpha
WT: wild-type
